# Improving the measurement of nitrogen stable isotopes in organic materials containing high C:N ratios using a 5A molecular sieve column

**DOI:** 10.1016/j.mex.2024.102889

**Published:** 2024-08-08

**Authors:** Matheus C. Carvalho, Paula Gomez-Alvarez, Luke C. Jeffrey, Damien Troy Maher

**Affiliations:** a Southern Cross Analytical Research Services, Southern Cross University, Lismore, NSW, Australia; b Faculty of Science and Engineering, Southern Cross University, Lismore, NSW, Australia

**Keywords:** 5A molecular sieve, Bark, Carbon monoxide, Elemental analysis, Nitrogen, Stable isotopes, 5A molsieve for δ^15^N

## Abstract

The nitrogen stable isotope composition (δ^15^N) of plant materials has numerous applications. Plant materials like bark can have a very high C:N ratio. Incomplete C combustion in such samples interferes with the δ^15^N measurement due to CO production. We modified the standard setup for δ^15^N measurement using an elemental analyzer (EA) coupled to an isotope ratio mass spectrometer (IRMS) by incorporating a 5A molecular sieve column, which better separates N_2_ from CO. We compared this new modified setup and the standard one for the measurement of bark samples. Precision and accuracy for δ^15^N in standards with low C:N ratio were equivalent for the two methods. However, for bark the results obtained with the new method had better precision and accuracy than the standard method. **Replicates are nevertheless recommended with the new method to ensure confidence in the results.**

• During elemental analysis, incomplete combustion of material with high C:N ratio can lead to CO formation, which interferes with δ^15^N IRMS measurements.
• Here we use a 5A molsieve column to remove the CO interference in δ^15^N measurements Precision and accuracy on δ^15^N measurements of samples with high C content are significantly improved

## Graphical abstract

**Figure:**
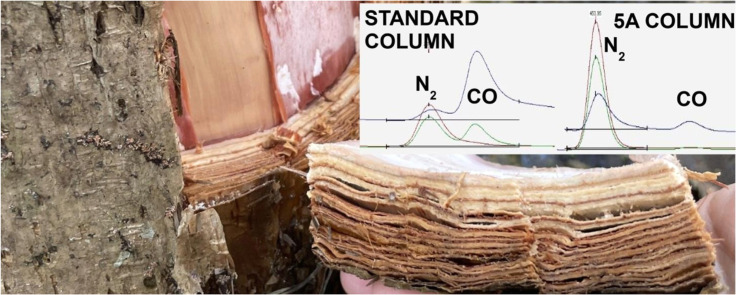

Specifications table

**Table:** 

Subject area:	Chemistry
More specific subject area:	*Stable isotope measurement*
Name of your method:	5A molsieve for δ^15^N
Name and reference of original method:	*Not applicable*
Resource availability:	*Not applicable*

## Background

In our laboratory, we have been employing the standard method to measure δ^15^N [1] for almost two decades. In this method, a sample of a solid powder is wrapped using tin foil, placed in an autosampler [2], and then combusted at 1020 °C in a combustion reactor. A pulse of oxygen is added at the time of the combustion to ensure a complete reaction, converting all carbon to CO_2_, and all nitrogen to NO or NO_2_. Helium flows constantly and carries the gases to a reduction reactor at 650 °C, where all nitrogen is reduced to N_2_. The He stream carries the resulting gases through a water trap to remove any traces of water, and a then through a Gas Chromatography (GC) column kept at 40 °C, where N_2_ and CO_2_ are separated from each other and from other potential trace gases, such as O_2_ which is used to aid combustion. The GC column used here is proprietary, that is, the manufacturer (Sercon) did not disclose its composition. However, such GC columns are usually made using Hayesep Q or Hayesep T meshes [3]. The gases are finally carried to an isotope ratio mass spectrometer (IRMS) where their stable isotope composition (δ^15^N for N, and δ^13^C for C) are measured. Stable isotope measurements are reported using the “delta notation”, in which the measured values are compared to internationally agreed reference values; the SI unit for values using the delta notation is Urey, usually expressed as milli Urey, or mUr. If only δ15N is necessary, a CO_2_ trap (e.g., soda lime) can be placed before the water trap, allowing for a shorter analysis time.

**Fig. 1:**
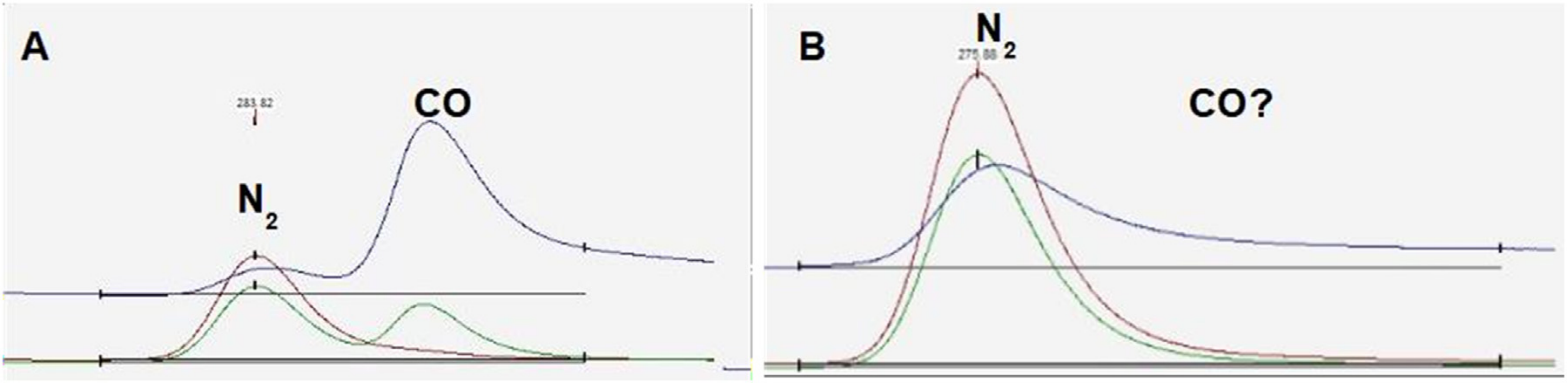
Chromatograms illustrating measurements of bark samples conducted using the standard setup, showing the presence of a CO peak alongside a N_2_ peak. A) The CO peak is clearly separated from the N_2_ peak. B) The CO peak is less distinct, although likely present. Despite the measured sample having a known δ^15^N value of 0.5mUr, the calculated values for Fig. 1A and Fig. 1B were 25.0 mUr and 7.6 mUr, respectively.

The method described above is robust and works for a large majority of organic samples. However, the measurement of δ^15^N in tree bark has been found to be problematic due to its high C:N ratio, which demands relatively large samples to be used in order to achieve higher precision in δ^15^N measurements. Nevertheless, this leads to an incomplete combustion of the C in the sample, and to the production of CO instead of CO_2_ [[4], [5], [6], [7]]. The CO_2_ trap does not work for CO, and the GC column does not separate CO from N_2_ with the same efficiency as it does for CO_2_. Consequently, a CO peak comes very close to the N_2_ peak, affecting the calculated δ^15^N values (Fig. 1A).

In some cases, however, visual inspection of the chromatogram does not always allow one to rule out CO interference on δ^15^N (Fig. 1B).

## Method details

**Fig. 2:**
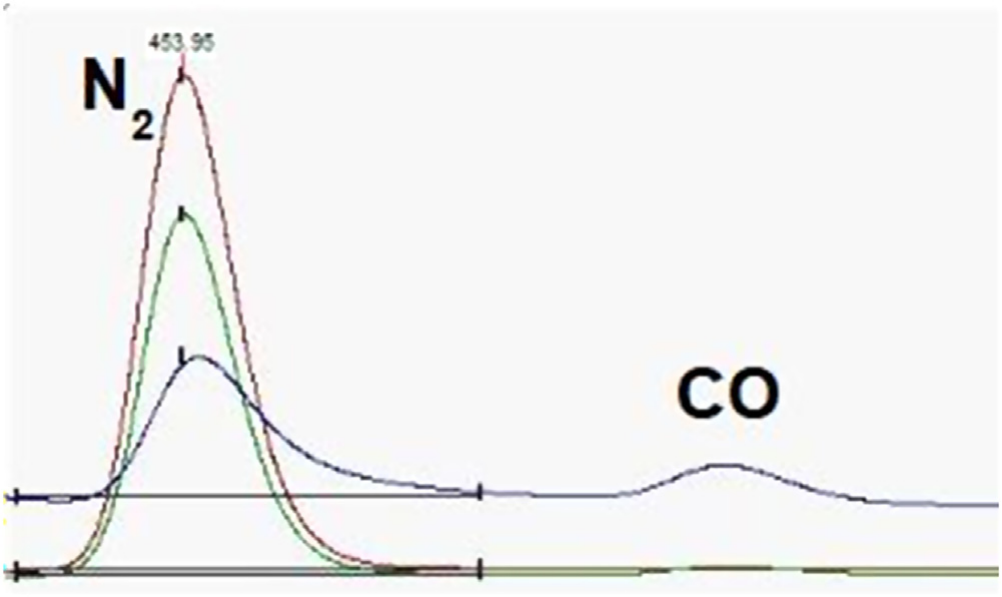
Chromatogram for the measurement of a bark sample performed using the modified setup, where a CO peak is present together with a N2 peak without interference. The measurement produced the expected δ^15^N value of 0.5 mUr.

Here we follow the standard method described above, with a small modification. When analyzing bark samples in our laboratory we replace the standard GC column (SC1996, by Sercon) with a 5A molsieve column (SC8428, by Sercon), which is known to better separate CO from N_2_. This way, even if a very small CO peak forms, it is separated from the N_2_ peak, and δ15N can be measured without interference (Fig. 2). The 5A molsieve column is kept at 40 °C during measurements.

## Method validation

### Example 1: Bark samples

The standard method was compared to the modified method for δ^15^N measurements using the bark of the wetland tree species *Melaleuca quinquenervia*. Bark samples where oven-dried at 60 °C for 4 days and subsequently homogenized by adding stainless steel beads (Qiagen) and employing a TissueLyser II (Qiagen) at 26 cycles/*sec* for one minute or more until finely powdered. In both cases, measurements were conducted for finely powdered bark samples (∼40 mg each), and measured alongside in-house standards (∼1 mg each) with known δ^15^N composition (glycine: δ^15^N = 2.0 mUr and caffeine: δ^15^N = −4.2 mUr), which had been determined by measuring them previously against international standards (USGS64: δ^15^N = 1.8 mUr and USGS65: δ^15^N = 20.7 mUr). Samples and standards were prepared using an autosampler [8], with amounts targeted to obtain peaks large enough to minimize blank influence (usually < 0.1 mUr). Measurements were conducted under identical conditions (oxidation reactor at 1020 °C, reduction reactor at 650 °C, soda lime to trap CO_2_, magnesium perchlorate to trap H_2_O, 30 s of O_2_ injection, measurement cycle lasting 800 s), except for the GC column used. For the standard method the SC1996 (by Sercon) GC column was utilized, while for the modified method the **SC8428** (by Sercon) GC column was used. Both columns were kept at 40 °C. In both treatments the samples were intercalated with empty cells in the autosampler, because previous experiments showed that consecutive bark measurements using SC8428 became unstable (results not shown).

Results for working standards were consistent for both methods (Table 1), indicating that both methods provided accurate and precise results for those substances. In contrast, results for bark samples obtained with the standard and modified methods presented significant differences (Table 1, *p* < 0.001), indicating that one (or both) of the methods was inaccurate. The standard method exhibited poor precision, and δ^15^N values could be very high (e.g., 104.6 mUr) when CO interference was strong, indicating its inaccuracy.

**Table 1:** Comparison between the standard and modified methods for the measurement of δ^15^N (unit: mUr; values shown as average ± standard deviation, followed by the number of measurements) in bark samples. P values are for unpaired Student *t* Test for means on a same table row.

Substance	Standard method	Modified method	P value
Glycine (working standard)	2.0 ± 0.08 *n* = 6	1.9 ± 0.13 *n* = 7	0.39
Caffeine (working standard)	−4.1 ± 0.08 *n* = 6	−4.1 ± 0.08 *n* = 8	0.79
Bark	28.6 ± 34.95 *n* = 21	0.4 ± 0.25 *n* = 21	0.001*

Significant values are marked with an * (α = 0.05).

Precision for bark samples using the modified method was notably improved (Table 1), and after removing four potential outliers, it matched the precision of the standards (0.4 ± 0.15 mUr, compare to Table 1). The three outliers had very similar values (0.0 ± 0.08 mUr), suggesting that they are not the result of poor homogenization, but rather some unaccounted-for uncertainty in the analytical procedure. Therefore, it is recommended that samples measured using this method are replicated, to account for potential less-than-optimum precision.

The accuracy of the modified method is supported by the improved precision for bark samples, and the precision and accuracy for the working standards (Table 1). Furthermore, the obtained average value for bark (0.5 mUr) is a common value for terrestrial plants [9].

### Example 2: Evaluating accuracy

The experiment with bark samples demonstrated that the modified method generated acceptable precision (Table 1) for both standards and bark samples. However, since the δ^15^N of the bark samples was initially unknown, the only evidence for its accuracy is that the obtained value is a common value with an acceptable precision. In order to explore possible deviations from accuracy with the modified method, mixtures of substances with known δ^15^N, such as cellulose, were prepared and measured. The proportions of the mixtures were determined to mimic the C:N ratio (∼300) of the bark. Only the modified method was used this time.

Although cellulose was expected to be N free, trace amounts of N were observed in it, which made necessary a blank correction for δ^15^N in the measurements using a mass balance. The δ^15^N in cellulose was found to be 14.6 ± 1.87 mUr (Table 2). The low precision was due to the small peak size. This value was used to obtain corrected δ^15^N values for the mixtures. The precision of the mixture measurements was lower than for pure substances due to this correction (Table 2), especially for caffeine. However, the average values for unmixed and mixed substances were very similar (Table 2), suggesting that the modified method yields accurate results.

**Table 2:** Measurement of δ^15^N in substances with known δ^15^N using the modified method. Data shown as in Table 1, except for P values that refer to unpaired Student *t-*test for averages between unmixed and mixed substances (glycine versus glycine plus cellulose; caffeine versus caffeine plus cellulose).

Substance	δ^15^N (mUr)	P value
Glycine	2.0 ± 0.04 *n* = 4	0.79
Glycine plus cellulose	2.1 ± 0.16 *n* = 4	
Caffeine	−4.2 ± 0.07 *n* = 4	0.91
Caffeine plus cellulose	−4.2 ± 0.86 *n* = 4	
Cellulose	14.6 ± 1.87 *n* = 3	

Significant values are marked with an * (α = 0.05).

## Limitations

The 5A column is useful for δ¹7 N measurements, but care should be taken if the CO₂ trap becomes exhausted. If so, the 5A column can trap CO₂ until its saturation, but then the column releases any new CO₂, making it impossible to do any measurement. Therefore, the column must be periodically baked between measurements to ensure that CO₂ or other impurities are expelled. In our experience, baking the column at 190 °C for 2 h after every 100 measurements worked well. Also, the 5A column cannot be used for δ¹tC measurements, as it traps CO₂ at 40 °C.

In summary, the application of the 5A molsieve column was successful for measuring δ^15^N in bark samples, and should work with other samples with a high C:N ratio. However, other analytical strategies could potentially achieve similar results. For example, the elemental analyzer commercialized by Elementar for δ^15^N uses a different analytical principle from that used here [10]. It is possible that their standard setup does not suffer from CO interference in the same way as described here.

## Ethics statements

None.

## CRediT author statement

MC: Conceptualization, Methodology, Validity tests, Manuscript writing. PA: Methodology, Validity tests, Manuscript writing. LJ: Methodology, Validity tests, Manuscript writing. DM: Conceptualization, Manuscript writing.

## Declaration of competing interest

The authors declare that they have no known competing financial interests or personal relationships that could have appeared to influence the work reported in this paper.

## Data Availability

Data will be made available on request.

## References

[1] Barrie A., Prosser S.J., Boutton T., Yamazaki S.-I. (1996). Mass Spectrometry of Soils.

[2] Carvalho M.C., Eickhoff W., Drexl M. (2020). Open-source autosampler for elemental and isotopic analyses of solids. HardwareX.

[3] Meier-Augenstein W. (2018).

[4] Savard M.M. (2020). Nitrogen isotopes of individual tree-ring series – The validity of middle- to long-term trends. Dendrochronologia.

[5] Couto-Vázquez A., González-Prieto S.J. (2014). Effects of biotic and abiotic factors on δ^15^N in young Pinus radiata. Eur. J. For. Res.

[6] Savard M.M., Marion J., Bégin C., Laganière J. (2023). On the significance of long-term trends in tree-ring N isotopes –The interplay of soil conditions and regional NOx emissions. Sci. Total Environ..

[7] Qi H., Coplen T.B., Jordan J.A. (2016). Three whole-wood isotopic reference materials, USGS54, USGS55, and USGS56, for δ^2^H, δ^18^O, δ^13^C, and δ^15^N measurements. Chem. Geol..

[8] Carvalho M.C. (2023). Automated weighing in the stable isotope lab: when less is more. MethodsX.

[9] Fry B. (2006).

[10] E.A. GmbH, Operating instructions vario MACRO cube CHNOS Elemental Analyzer. (2017)

